# Effects of optimizing emergency nursing care process on success rate of rescue and incidence of adverse reactions in patients with acute chest pain

**DOI:** 10.3389/fmed.2025.1727151

**Published:** 2026-01-08

**Authors:** Hongyan Zhu, Dongsheng Ding

**Affiliations:** Department of Emergency Room, Suzhou Hospital of Integrated Traditional Chinese and Western Medicine, Suzhou, Jiangsu, China

**Keywords:** acute chest pain, adverse reactions, optimizing emergency nursing care process, rescue, workflow optimization

## Abstract

**Objective:**

To explore the impacts of optimizing emergency nursing care process on success rate of rescue and incidence of adverse reactions in patients with acute chest pain.

**Methods:**

A total of 90 emergency patients with acute chest pain, admitted between January 2022 and December 2023, were selected as study participants. Patients were randomly assigned to either the control group or the observation group using a random number table method. The control group received routine care, while the observation group underwent an optimized emergency nursing care process. Comparisons between the two groups were made for the following outcomes: rescue and hospitalization time, rescue success rate, pain intensity, incidence of adverse reactions, mental status, and nursing satisfaction.

**Results:**

Compared with the control group, the rescue and hospitalization time of the observation group were shorter (*p* < 0.01). Compared with the control group, the rescue success rate of the observation group was higher (82.22% vs. 97.78%; *p* = 0.013). The visual analogue score (VAS) scores of the observation group at 30 min, 60 min, 120 min and 240 min after rescue were lower than those of the control group (*p* < 0.01). Compared with the control group, the incidence of adverse reactions of the observation group was lower (20.00% vs. 4.44%; *p* = 0.024). Compared with the control group, the self-rating anxiety scale (SAS) and self-rating depression scale (SDS) scores of the observation group were lower after nursing (*p* < 0.01). Compared with the control group, the nursing satisfaction score of the observation group was higher (*p* < 0.01).

**Conclusion:**

The optimized emergency nursing care process for patients with acute chest pain is associated with relief of pain, a decrease in the occurrence of negative psychology and adverse reactions, as well as an effective improvement in the success rate of rescue.

## Introduction

The emergency department is the main place for treating critically ill patients. Various diseases are common in the emergency department, among which acute chest pain is one of the common symptoms in critically ill patients (1). Acute chest pain is more common in patients with myocardial infarction, myocarditis, coronary artery syndrome, angina pectoris, vertebral artery dissection, and other diseases. These diseases have the characteristics of rapid onset and rapid progression (2). In recent years, with the acceleration of population aging in our country, the incidence of acute cardiovascular diseases has increased, and the number of patients visiting hospitals due to acute chest pain has also increased year by year (3). Patients with acute chest pain presents various clinical manifestations and progresses rapidly, requiring highly dependent treatment timing. If patients fail to receive timely treatment, they will face the risk of death (4). Therefore, the emergency department should immediately identify the causes of the patient’s chest pain and take effective treatment measures promptly to further improve the clinical rescue success rate and help patients improve their prognosis.

Emergency nursing is an important part of the emergency department. The quality of emergency nursing directly affects the quality of life of patients and the overall quality of medical services (5). In the routine emergency nursing work, there are still some problems and deficiencies, such as unclear nursing work processes, insufficient system integrity, and lack of standardization in the execution process (6). These may increase the risk of errors, reduce the success rate of rescue, and even cause damage to the patient’s health condition (7). Therefore, optimizing the emergency nursing care process is of great significance for improving the quality of emergency nursing care and the success rate of rescue operations. Some scholars have pointed out that optimizing the emergency nursing care process can significantly improve the rescue success rate of patients (8). For instance, Wei et al. suggested that the adopting an optimized emergency nursing care process for patients with hepatic encephalopathy is effective, which effectively improves the success rate of hepatic encephalopathy resuscitation and improves the prognosis of patients (9).

Therefore, our study analyzed the impacts of optimizing emergency nursing care process on success rate of rescue and incidence of adverse reactions in acute chest pain patients, aiming to provide emergency nursing care reference for patients with acute chest pain.

## Materials and methods

### General data

**Figure 1:**
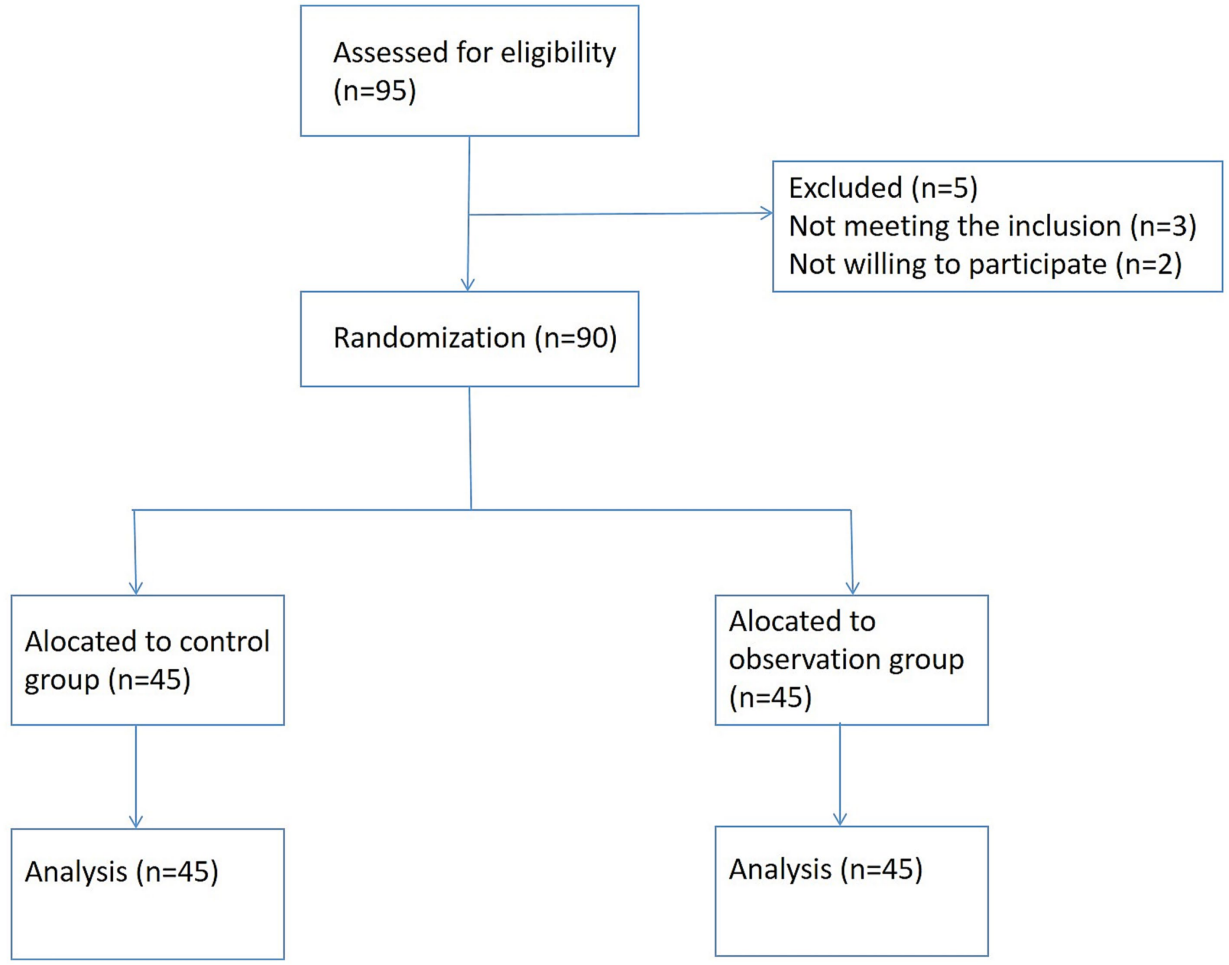
CONSORT-style flow diagram.

Ninety emergency patients with acute chest pain admitted to our hospital from January 2022 to December 2023 were chosen to be the study participants. Patients were randomized into control and observation groups (*n* = 45 each) using a random number table method, and the flow diagram was shown in Figure 1.

Inclusion criteria: (1) Patients met the diagnostic criteria for acute chest pain caused by acute coronary syndrome (ACS), acute myocarditis, angina, aortic dissection acute myocardial infarction. For ACS, patients had acute chest pain with characteristics like pressure or tightness, along with electrocardiograph (ECG) changes (e.g., ST-segment elevation/depression) and elevated cardiac troponin levels. In acute myocarditis, chest pain, often sharp, was accompanied by ECG abnormalities (e.g., diffuse ST-segment elevation) and confirmed by cardiac magnetic resonance imaging (MRI) showing myocardial inflammation. Angina patients experienced exertion-triggered chest pain relieved by rest, with ECG changes during episodes. Aortic dissection patients presented with sudden, severe, tearing chest pain, and diagnosis was confirmed by imaging showing the dissection flap. Acute myocardial infarction patients had chest pain similar to ACS, significant ECG changes, and a marked rise/fall in troponin. (2) All patients and their families were aware of the content of this study and had voluntarily signed informed consent; Exclusion criteria: (1) Patients had heart, liver, kidney and other vital organ diseases; (2) Patients had mental and conscious disorders; (3) Incomplete clinical data; (4) Patients with myocarditis, pericarditis and pleural chest pain.

This study was conducted in accordance with the Declaration of Helsinki (1964) and its subsequent amendments. Ethical approval for the study was obtained from the Ethics Committee of Suzhou Hospital of Integrated Traditional Chinese and Western Medicine on January 5, 2022, and the approval number was 2022-01SZ-LL003. Written informed consent was obtained from all participants. The Clinical Registration Number was ChiCTR2100051587.

### Sample size

The sample size and power calculations were performed using G*Power 3.1 software. An alpha level of 0.05 and a power of 0.85 were set to control type I and type II errors, respectively. We planned to use an independent-samples t-test to compare the two groups. The assumed effect size was set at Cohen’s d = 0.6, which was based on the medium effect size value derived from previous studies on rescue time (10). Based on these parameters, G*Power 3.1 determined that a total sample size of 90 (45 participants per group) was adequate to detect the assumed effect size with the specified power and alpha level, validating the sample size for our study.

Given an alpha level of 0.05 and a power of 0.85, two groups were generated with a total sample size of 90, with 45 participants per group.

### Randomization and blinding

A group randomization design with a 1:1 allocation ratio was used to assign eligible patients to either the control group or the observation group. The random allocation sequence was generated by an independent statistician using computer-generated random numbers (R software, version 4.2.0). After generating the randomization sequence, allocations were placed in pre-prepared, sealed, opaque envelopes labeled with consecutive study IDs. Envelopes were stored in a locked cabinet accessible only to the research coordinator, who was not involved in patient enrollment or intervention delivery. When a patient met inclusion criteria, the research coordinator opened the next envelope in sequence to reveal the group assignment and notified the nursing team.

The study was single-blinded, with patients unaware of their group assignment to minimize reporting bias. During consent, patients were informed they would receive “routine nursing care or optimized emergency nursing care” but not told which group they were in. Intervention nurses (delivering optimized care) were not blinded to group assignment due to the nature of the nursing process modifications. To mitigate performance bias, outcome assessors were blinded to group allocation. These assessors did not participate in intervention delivery.

### Methods

#### Control group

The control group received routine emergency nursing care aligned with institutional guidelines, structured as follows:

##### Initial triage and rescue pathway activation

###### Triage assessment

Upon admission, nurses conducted a primary triage based solely on the patient’s chief complaint (e.g., chest pain, dyspnea) using a 5-level emergency severity index (ESI).

Patients classified as ESI Level 1 or 2 (highest acuity) were immediately prioritized for rescue.

###### Rescue pathway initiation

Nurses activated the standard rapid rescue pathway, which included:

Transfer to the emergency resuscitation room within 3 min.

Attachment to a 12-lead ECG monitor within 5 min of arrival.

Notification of the attending physician via pager system for immediate evaluation.

##### Diagnostic and therapeutic interventions

###### Venous access and blood sampling

A peripheral intravenous catheter was inserted within 5 min of arrival for fluid/medication administration.

Blood samples were collected for routine tests (complete blood count, coagulation profile) and acute cardiac markers (troponin I, CK-MB), with results reported within 30 min.

###### Pharmacological treatment

Medications (e.g., aspirin, nitroglycerin, anticoagulants) were administered only after physician orders, typically within 10 min of doctor assessment.

##### Surgical preparation and coordination

###### Preoperative readiness

For patients requiring emergency PCI (percutaneous coronary intervention) or surgery:

Nurses completed standard preoperative checks (e.g., allergy history, consent forms) but did not initiate advanced preparations (e.g., preoperative antibiotics, Foley catheter insertion) without explicit physician orders.

###### Intraoperative support

During procedures, nurses followed routine monitoring protocols (e.g., vital signs every 5 min) and provided passive assistance (e.g., handing instruments) under surgeon direction.

#### Observation group

The observation group received optimized emergency nursing care, and the specific methods were as follows:

##### Chest pain specialist nursing team setup

###### Team composition

Team Leader: 1 senior emergency nurse (≥5 years of experience) responsible for oversight and coordination.

Core members: 4–6 nurses (including 2 with advanced cardiac life support certification) and 1 nurse technician.

Support roles: 1 physician advisor (cardiologist) and 1 IT specialist for system maintenance.

###### Training components

Theoretical courses:

High-risk chest pain identification [e.g., using the History, Electrocardiogram, Age, Risk Factors, Troponin (HEART) score or Thrombolysis in Myocardial Infarction (TIMI) risk score].

ECG interpretation [focus on ST-segment elevation myocardial infarction (STEMI), non-STEMI (NSTEMI), and arrhythmias].

Rapid bedside troponin testing protocols.

###### Skill workshops

Simulation training for triage, oxygen therapy, and venous access establishment.

Use of electronic pre-registration systems and computer-assisted triage tools.

Certification: All nurses completed 16 h of training and passed a competency exam (score ≥85%) prior to implementation.

##### Streamlined triage protocol

###### Workflow

Initial assessment: Triage nurse uses a computerized pre-check system to evaluate patients within 3 min of arrival.

Risk stratification:

High-risk criteria:

STEMI on ECG.

Hemodynamic instability [systolic blood pressure (SBP) < 90 mmHg, heart rate (HR) > 120 bpm].

Severe chest pain [Visual Analogue Scale (VAS) ≥ 8] unresponsive to nitroglycerin.

###### Low-risk criteria

Normal/non-specific ECG.

Stable vital signs and mild pain (VAS < 4).

###### Disposition

High-risk: Immediate escort to the rescue room with activation of the code STEMI protocol (e.g., notifying cardiology, cath lab team).

Low-risk: Transfer to the chest pain observation unit for ECG (within 10 min) and serial troponin testing (at 0 and 3 h).

##### Green channel for expedited care

###### Key measures

Priority testing: All patients undergo ECG within 10 min of arrival, followed by troponin testing [bedside point-of-care testing (POCT) device, result in ≤20 min].

Imaging coordination: For suspected aortic dissection/pulmonary embolism, nurses accompany patients to computerized tomography (CT)/X-ray and prioritize scans. Charges are processed post-stabilization.

Documentation: A standardized electronic form captures triage data, test results, and interventions in real time.

##### Rescue nursing procedures

High-risk patients:

Immediate actions:

Oxygen therapy (target SpO₂ ≥ 95%).

Monitor attachment (ECG, SBP, SpO₂).

IV access (2 large-bore catheters) and blood draw for labs.

###### Medication administration

Aspirin 300 mg chewable + clopidogrel 300 mg loading dose (for ACS).

Nitroglycerin (sublingual/IV) for ongoing chest pain.

###### Advanced care

Prepare for thrombolysis or percutaneous coronary intervention (PCI; e.g., anticoagulants, heparin bolus).

For aortic dissection: IV beta-blockers (e.g., esmolol) to reduce dP/dt.

###### Low-risk patients

Monitor in the observation unit (q15min vitals, repeat ECG if symptoms worsen).

Discharge education on medication adherence and warning signs of complications.

##### Symptom-specific interventions

ACS (STEMI/NSTEMI): Administer dual antiplatelet therapy, prepare for PCI/thrombolysis, monitor for arrhythmias.

**Table:** 

Diagnosis	Nursing actions
ACS (STEMI/NSTEMI)	Administer dual antiplatelet therapy, prepare for PCI/thrombolysis, monitor for arrhythmias.
Aortic dissection	Sedation (morphine), BP control (IV labetalol), avoid Valsalva maneuvers.
Pulmonary embolism	Oxygen therapy, anticoagulation (Low Molecular Weight Heparin), prepare for catheter-directed thrombolysis.
Musculoskeletal pain	Heat therapy, analgesics (e.g., acetaminophen), reassurance.

##### Psychological support

###### Communication strategy

For conscious patients:

Use the BATHE protocol (Background, Affect, Trouble, Handling, Empathy) to assess emotional state.

Provide clear explanations of procedures (e.g., “The ECG will help us rule out a heart attack”).

For family members:

Designate a nurse to update them on the patient’s status and treatment plan.

##### Process monitoring and quality control

###### Audit tools

Weekly review of triage accuracy, door-to-ECG times, and medication errors.

Monthly team debriefings to address gaps (e.g., delays in troponin testing).

###### Performance metrics

Primary: Door-to-balloon time (for STEMI), rescue success rate.

Secondary: Patient satisfaction (5-point Likert scale), unplanned readmissions.

#### Observation indicators

Rescue and hospitalization time, rescue success rate, degree of pain, incidence of adverse reactions (shock, stroke, arrhythmia, and heart failure), mental status and nursing satisfaction of patients were compared in 2 groups. Rescue success rate: Defined as successful restoration of vital signs (stable hemodynamics, SpO_2_ ≥ 95%, and resolution of acute symptoms within 24 h post-intervention) without progression to life-threatening complications (e.g., cardiac arrest, shock). Visual analogue score (VAS) was compared between 2 groups at 30 min, 60 min, 120 min along with 240 min after rescue (11). The total score was 10 points. The higher score represented higher degree of pain. The VAS was measured as follows: Nurses used a vernier scale about 10 cm long, marked with 10 scales on one side, with a “0” end and a “10” end at each end, where 0 indicated no pain, 10 indicated severe pain, and the middle part indicated varying degrees of pain. The patient was asked to mark the level of pain on a line based on how they feel, with a score of 1–3 representing mild pain, 4–6 moderate pain, and 7–10 severe pain. The Self-rating Anxiety Scale (SAS) and Self-rating Depression Scale (SDS) were adopted to evaluate the anxiety and depression of patients (12). The higher scores represented higher levels of anxiety and depression. The nursing satisfaction score was calculated by the self-made questionnaire of the department. The total score of the questionnaire is the sum of the scores from all 10 questions, ranging from a minimum of 10 points to a maximum of 50 points. To convert it to a 100-point scale, multiply the total score by 2. Higher scores indicate higher levels of nursing satisfaction. The scale validity coefficient of this questionnaire is 0.784, which indicates a relatively high degree of accuracy in measuring the intended concept of nursing satisfaction. The reliability coefficient is 0.865, suggesting that the questionnaire has good internal consistency and can produce stable and reliable results when administered multiple times under similar conditions.

### Statistical analysis

Statistical analysis was performed using SPSS 24.0. For categorical variables, which were presented as frequencies and percentages [n (%)], and χ^2^ test was used to compare the differences between the two groups. For continuous variables, which were presented as the mean ± standard deviation (x̅±s), we first used the Shapiro–Wilk test to assess the normality of the data distribution within each group. If the data in both groups followed a normal distribution, we proceeded with the independent samples t-test. If the data in both groups did not follow a normal distribution, we used the Mann–Whitney U test to compare the differences between the two groups. Multiple comparisons between different groups were analyzed by two-way ANOVA, and 95% confidence intervals (95% CI) were provided. *p* < 0.05 was considered statistically significant.

## Results

### General data of patients in 2 groups

No differences were seen in general data of patients between 2 groups (*p* > 0.05), reflecting comparability (Table 1).

**Table 1:** General data of patients in 2 groups (mean ± standard deviation)/[*n* (%)].

Items	Control (*n* = 45)	Observation (*n* = 45)	t/χ^2^	*p*
Gender			0.178	0.672
Male	25 (55.56)	23 (51.11)		
Female	20 (44.44)	22 (48.89)		
Age (years)	43.36 ± 7.56	43.42 ± 7.64	0.037	0.970
Causes of chest pain			0.196	0.978
Acute myocarditis	11 (24.44)	12 (26.67)		
Angina	7 (15.56)	6 (13.33)		
Aortic dissection	12 (26.67)	11 (24.44)		
Acute myocardial infarction	15 (33.33)	16 (35.56)		
Time from onset to visit (h)	12.02 ± 0.94	12.08 ± 1.05	0.285	0.775
Heart rate (bpm)	85.25 ± 10.53	86.10 ± 9.87	0.395	0.693
Systolic blood pressure (mmHg)	130.54 ± 15.23	132.37 ± 14.71	0.579	0.563
Diastolic blood pressure (mmHg)	78.69 ± 10.13	79.22 ± 9.65	0.254	0.800
History of diabetes	12 (26.67)	14 (31.11)	0.216	0.641
History of hypertension	20 (44.44)	21 (46.67)	0.044	0.832
Baseline SAS score (points)	62.85 ± 6.28	62.89 ± 6.32	0.030	0.976
Baseline SDS score (points)	61.78 ± 6.18	61.83 ± 6.24	0.038	0.969

### Rescue and hospitalization time in 2 groups

**Figure 2:**
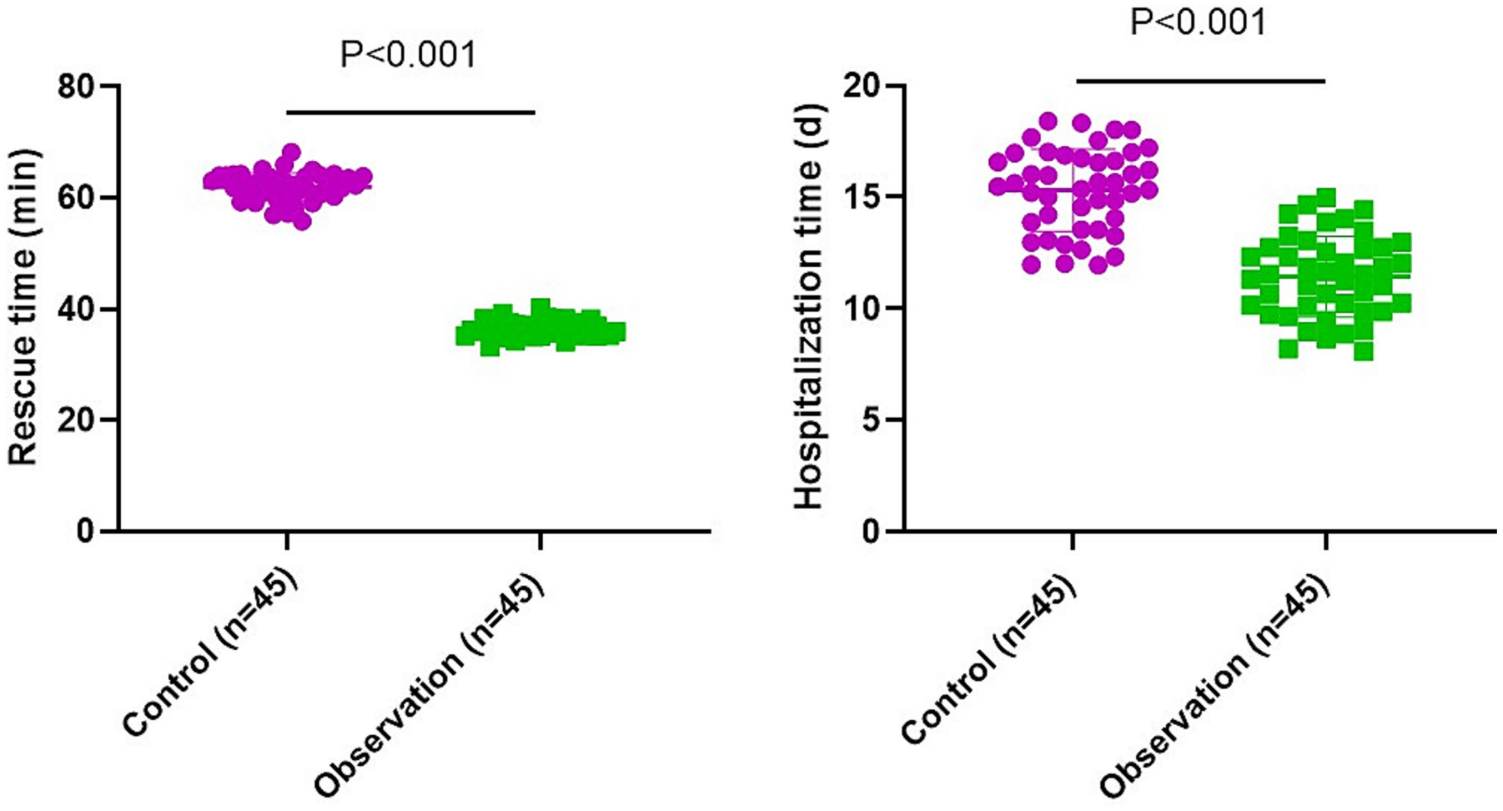
Rescue and hospitalization time in 2 groups.

The observation group had shorter rescue and hospitalization time compared to the control group (*p* < 0.001, *t* = 60.29, df = 44, 95% CI: −26.42–−24.73; *p* < 0.001, *t* = 10.03, df = 44, 95% CI: −4.637–−3.103; Figure 2).

### Rescue success rate in 2 groups

The observation group had better success rate of rescue compared to the control group (*p* = 0.013, χ^2^ = 6.049, df = 1, 95% CI: 0.381–0.841; Table 2).

**Table 2:** Rescue success rate in 2 groups [n (%)].

Groups	N	Number of successful rescue	Number of unsuccessful rescue
Control	45	37 (82.22)	8 (17.78)
Observation	45	44 (97.78)	1 (2.22)
χ^2^			6.049
*p*			0.013
95% CI			0.381–0.841

### VAS score in 2 groups

**Figure 3:**
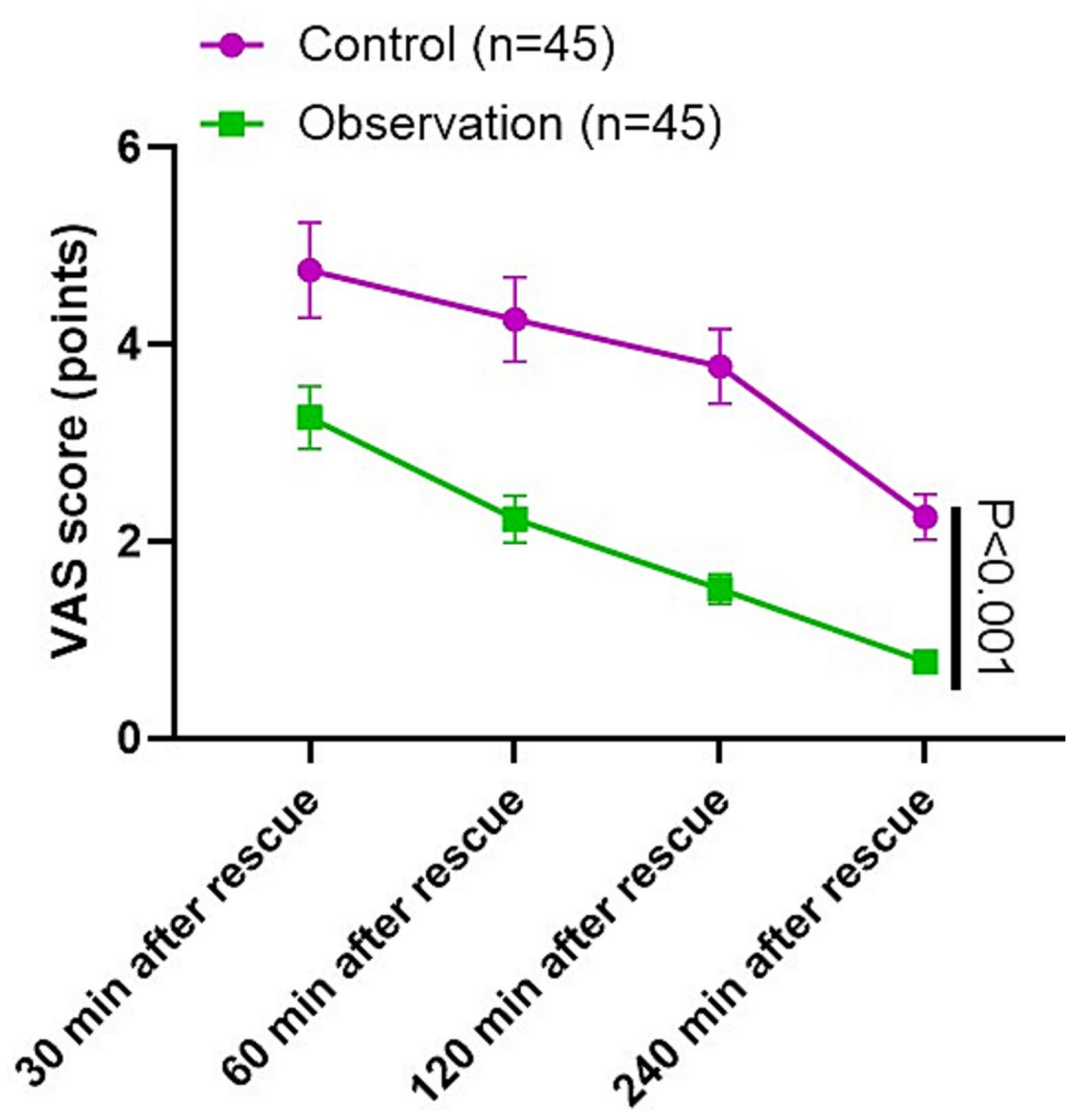
VAS score in 2 groups.

As Figure 3 shown, the VAS scores of the observation group at 30 min, 60 min, 120 min and 240 min after rescue were lower compared to the control group (*p* < 0.001; *F* = 34.62, df = 3, 95% CI: 1.749–1.881).

### Incidence of adverse reactions in 2 groups

The observation group had lower incidence of adverse reactions compared to the control group (*p* = 0.024, χ^2^ = 5.075, df = 3, 95% CI: 1.097–2.484; Table 3).

**Table 3:** Incidence of adverse reactions in 2 groups [n (%)].

Groups	N	Shock	Stroke	Arhythmia	Heart failure	Total incidence rate
Control	45	3 (6.67)	1 (2.22)	3 (6.67)	2 (4.44)	9 (20.00)
Observation	45	1 (2.22)	0 (0.00)	1 (2.22)	0 (0.00)	2 (4.44)
χ^2^						5.075
*p*						0.024
95% CI						1.097–2.484

### Mental status of patients in 2 groups

**Figure 4:**
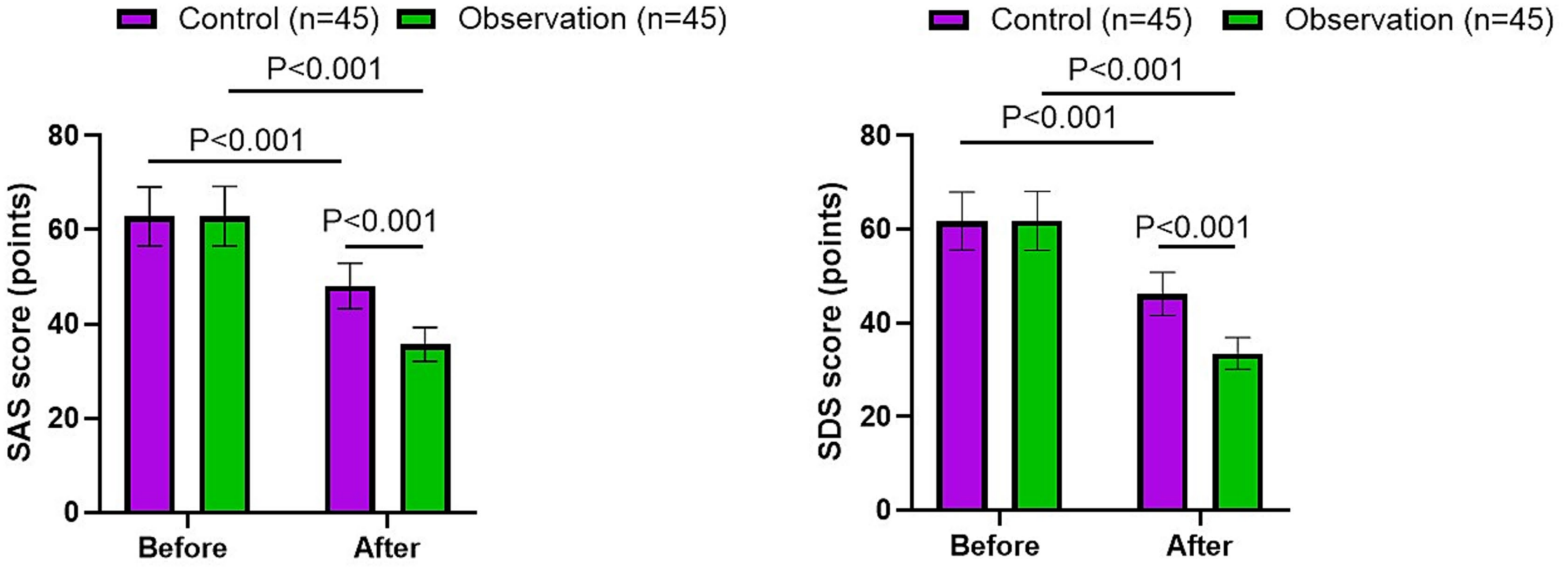
Mental status of patients in 2 groups.

As Figure 4 shown, after nursing, the SAS and SDS scores were declined in 2 groups (*p* < 0.001, *F* = 59.46, df = 1, 95% CI: 4.595–7.755; *p* < 0.001, *F* = 65.81, df = 1, 95% CI: 4.794–7.876;), and the observation group had lower SAS and SDS scores compared to the control group (*p* < 0.001, *F* = 685.4, df = 1, 95% CI: 19.38–22.55; *p* < 0.001, *F* = 789.0, df = 1, 95% CI: 20.39–23.48).

### Nursing satisfaction of patients in 2 groups

**Figure 5:**
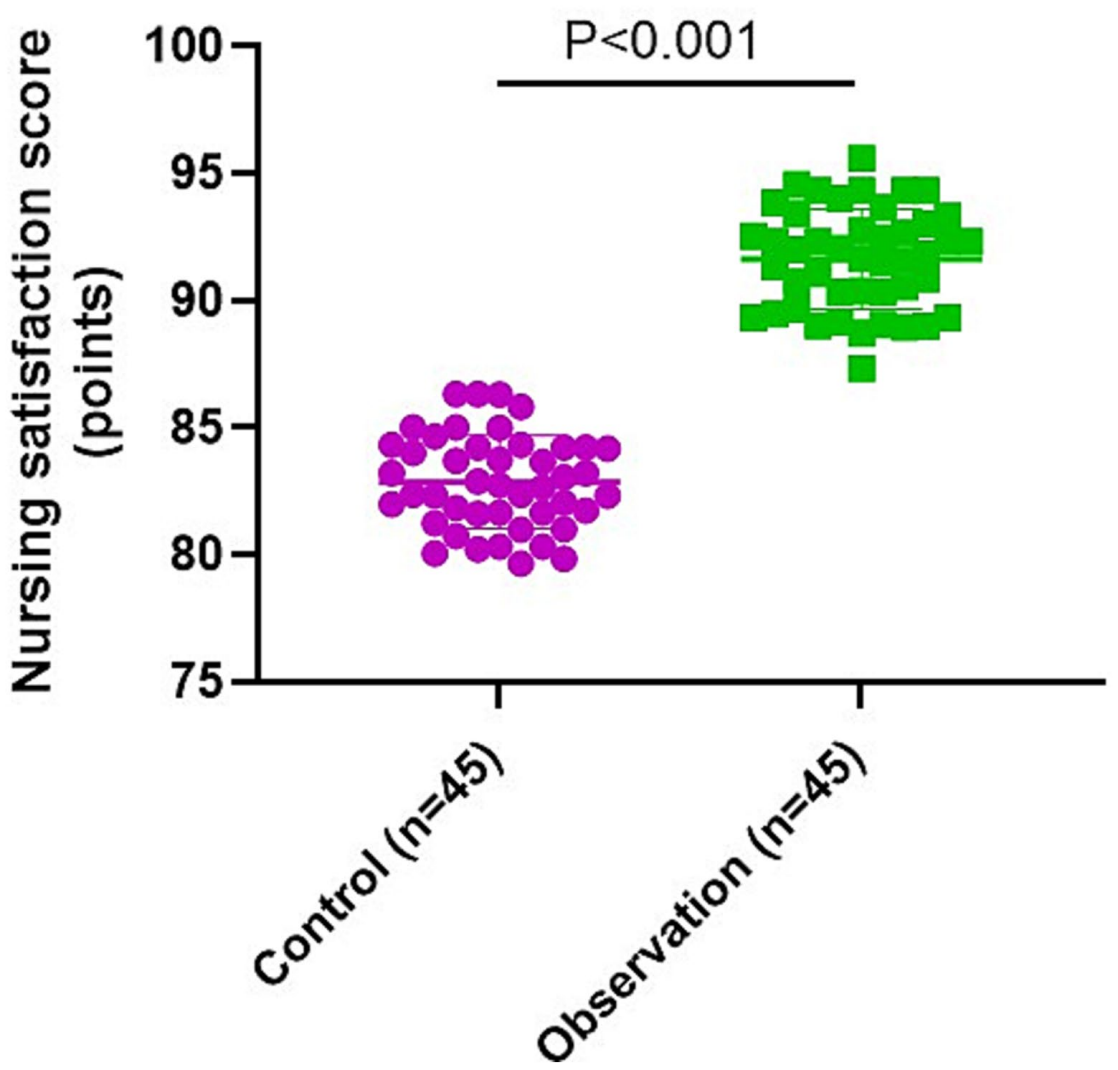
Nursing satisfaction of patients in 2 groups.

The observation group had higher nursing satisfaction score compared to the control group (*p* < 0.001, t = 21.89, df = 44, 95% CI: 7.970–9.562; Figure 5).

## Discussion

Chest pain, as a symptom that poses a serious threat to the life safety of patients, is a condition that is severe and has complex causes (13). Timely implementation of scientific and effective treatment measures is crucial for improving the prognosis of patients with chest pain. During the emergency treatment of patients with chest pain, emergency nursing care is of vital importance. High-quality emergency care not only shortens the emergency hospital stay of patients but also further increases the success rate of rescue (14).

In the past, the routine emergency nursing case process was mainly carried out according to the instructions of doctors. This not only led to an unstandardized care process, but also resulted in unclear care goals and low care quality, thereby greatly restricting the improvement of the success rate and efficiency of patient rescue (15). Recently, with the continuous deepening of clinical research on emergency nursing care for patients with acute chest pain, the routine emergency nursing care process has been optimized to a certain extent (16). Clinical practice has shown that the optimized emergency nursing care process has a positive effect on improving the rescue success rate of patients with acute chest pain. It can ensure that the diagnosis, transfer and initial emergency treatment of patients can be carried out quickly and smoothly during the emergency period (17).

In this study, the results displayed that compared with the control group, the observation group had shorter rescue and hospitalization time, better success rate of rescue, lower VAS score, lower incidence of adverse reactions as well as higher nursing satisfaction score. One possible reason is that the optimized emergency nursing care process could clearly define the job responsibilities and tasks of different nurses, enabling them to acquire the necessary professional knowledge and skills for their respective positions, which may effectively improve the quality of care. During the nursing process, the nurse quickly conducted a preliminary examination and classification of the patient’s condition, completed all the examinations for the patient promptly, and promptly sent the patient to the emergency room for emergency treatment. These streamlined steps effectively ensured the timeliness and relative effectiveness of the rescue treatment, thereby reducing the time spent on rescue and hospitalization.

The optimized process also contributed to faster diagnosis. By having a well-defined and coordinated approach, nurses were able to gather relevant patient information more efficiently and communicate it effectively to the medical team. This facilitated a more rapid and accurate assessment of the patient’s condition, leading to a quicker diagnosis. For example, in the case of patients with acute chest pain, early and accurate diagnosis is crucial for initiating the appropriate treatment promptly. The optimized emergency nursing care process may have played a key role in achieving this faster diagnosis, which in turn was associated with improved patient outcomes.

Effective communication among the healthcare team is essential for the successful treatment of patients with acute chest pain. The optimized emergency nursing care process likely enhanced communication between nurses and doctors, as well as among different nursing staff. Clear job responsibilities and a standardized workflow ensured that everyone was on the same page regarding the patient’s care plan. This improved communication may have led to more coordinated and efficient care delivery, reducing the likelihood of errors or misunderstandings that could delay treatment.

At the same time, compared with the control group, the observation group had lower SAS and SDS scores after nursing. One possible explanation is that nurses in the optimized process paid close attention to assessing and caring for the patients’ psychological states. By providing emotional support and reassurance, they effectively alleviated the patients’ negative emotions. This, in turn, enabled patients to cooperate more actively with the rescue treatment and nursing work. When patients are in a more positive psychological state, they are more likely to follow medical advice, participate in treatment procedures, and tolerate potential discomfort, all of which can contribute to a higher success rate of rescue.

The American Heart Association (AHA) and the European Society of Cardiology (ESC) have established guidelines for the management of chest pain centers. These guidelines emphasize the importance of a standardized and efficient workflow in the emergency care of patients with chest pain. They recommend the use of risk stratification tools to quickly identify high-risk patients and initiate appropriate treatment promptly (18, 19). Our optimized emergency nursing care process aligns with these international guidelines by incorporating a preliminary examination and classification of the patient’s condition, which is in line with the risk stratification approach advocated by the AHA and ESC.

Several workflow optimization frameworks have been proposed globally to improve the emergency care of patients. For example, the Lean methodology, which focuses on eliminating waste and improving efficiency, has been applied in healthcare settings (20). Our optimized emergency nursing care process can be seen as a local adaptation of such workflow optimization principles. By clearly defining job responsibilities and streamlining the care process, we have reduced unnecessary steps and improved the overall efficiency of emergency care, similar to the goals of Lean-based workflow optimization.

Previous studies have also shown the benefits of optimized emergency care processes. Li et al. suggested that using a “four-in-one” optimized emergency nursing care process, the treatment time in the emergency room of mushroom poisoning patients was significantly shortened, and nursing satisfaction was improved (21). Shen et al. indicated that multidisciplinary collaborative nursing process enhanced improved the prognosis of patients with hypertensive cerebral hemorrhage (22).

Our study has some limitations. First, the trial was conducted in a single center, which may limit the generalizability of our findings to a broader patient population. Second, the number of patients included in this study was small, reducing the statistical power and potentially increasing the risk of Type II errors (failing to detect a true effect when one exists). Third, our outcome measures are mostly subjective rather than objective, which could introduce bias into the results and affect the reliability of our conclusions. Moreover, it is important to note that there are no adjustments for multiple comparisons in our statistical analysis. When multiple hypotheses are tested simultaneously, the probability of obtaining a false-positive result (Type I error) increase. Without proper adjustments, such as the Bonferroni correction or false discovery rate control methods, the significance levels reported for our findings may be overestimated, leading to potentially misleading conclusions. Furthermore, the description of the intervention measures in our article is not detailed enough, which may affect the reproducibility of the intervention measures in clinical practice. Therefore, more detailed, large-scale, multi-center, and objective studies with appropriate adjustments for multiple comparisons are needed in the future to confirm and expand upon our results.

## Conclusion

Our study demonstrates that the optimized emergency nursing care process for patients with acute chest pain is associated with relief of pain, a decrease in the occurrence of negative psychology and adverse reactions, as well as an effective improvement in the success rate of rescue. This process may be worthy of further exploration and promotion in clinical practice and could provide a reference for the emergency care of patients with acute chest pain in a global context, considering international guidelines and previous research findings.

## Data Availability

The datasets presented in this study can be found in online repositories. The names of the repository/repositories and accession number(s) can be found in the article/Supplementary material.
