# Supplementary material for: Effects of optimizing emergency nursing care process on success rate of rescue and incidence of adverse reactions in patients with acute chest pain
Source: Front Med (Lausanne). 2026 Jan 8;12:1727151. doi: 10.3389/fmed.2025.1727151 (PMC12823961; doi:10.3389/fmed.2025.1727151)
Supplement: Supplementary file 1 [file Table_1.DOCX]

**Self-made Nursing Satisfaction Questionnaire for Patients with Acute Chest Pain**

| Section | Question Content | Response Options | Score Assignment |
| --- | --- | --- | --- |
| I Nursing Attitude and Communication | 1. How would you rate the overall friendliness and politeness of the nursing staff during your emergency care? | 1 - Very unfriendly 2 - Unfriendly 3 - Neutral 4 - Friendly 5 - Very friendly | 1 point for each option, with a maximum of 5 points for this question. |
|  | 2. Did the nurses communicate with you in a clear and understandable manner? | 1 - Not at all clear 2 - Slightly clear 3 - Moderately clear 4 - Quite clear 5 - Extremely clear | 1 - 5 points corresponding to the options. |
|  | 3. Were the nurses willing to listen to your concerns and answer your questions? | 1 - Never 2 - Rarely 3 - Sometimes 4 - Often 5 - Always | 1 - 5 points for each response. |
| II. Nursing Skill and Efficiency | 4. How satisfied are you with the speed at which the nurses responded to your emergency situation? | 1 - Very dissatisfied  2 - Dissatisfied  3 - Neutral  4 - Satisfied  5 - Very satisfied | 1 - 5 points based on the level of satisfaction. |
|  | 5. Did the nurses perform their nursing procedures (e.g., intravenous injection, monitoring) skillfully? | 1 - Very unskillfully 2 - Unskillfully 3 - Moderately skillfully 4 - Skillfully 5 - Very skillfully | 1 - 5 points for each option. |
|  | 6. Were the nurses able to coordinate well with other medical staff during your care? | 1 - Poorly coordinated 2 - Somewhat poorly coordinated 3 - Moderately coordinated 4 - Well coordinated 5 - Extremely well coordinated | 1 - 5 points according to the coordination level. |
| III. Environmental and Comfort Factors | 7. How would you rate the cleanliness of the emergency care area? | 1 - Very dirty 2 - Dirty 3 - Moderately clean 4 - Clean 5 - Very clean | 1 - 5 points for each cleanliness level. |
|  | 8. Was the emergency care environment quiet enough for you to rest and recover? | 1 - Very noisy  2 - Noisy  3 - Moderately quiet  4 - Quiet  5 - Very quiet | 1 - 5 points depending on the noise level. |
|  | 9. Did the nursing staff provide you with appropriate comfort measures (e.g., adjusting the bed position, providing blankets)? | 1 - Never provided 2 - Rarely provided 3 - Sometimes provided 4 - Often provided 5 - Always provided | 1 - 5 points for each frequency option. |
| IV. Overall Satisfaction | 10. Overall, how satisfied are you with the emergency nursing care you received? | 1 - Very dissatisfied 2 - Dissatisfied 3 - Neutral 4 - Satisfied 5 - Very satisfied | 1 - 5 points based on overall satisfaction. |
